# Psycho-physical interactions in Parkinson’s Disease: Protocol for a prospective observational cohort study

**DOI:** 10.1371/journal.pone.0315345

**Published:** 2024-12-16

**Authors:** Philip Hodgson, Alastair Jordan, Charikleia Sinani, Divine Charura

**Affiliations:** 1 Physiotherapy Department, Tees, Esk and Wear Valleys NHS Foundation Trust, West Park Hospital, Darlington, United Kingdom; 2 School of Science, Technology and Health, York St John University, York, United Kingdom; 3 School of Education, Language and Psychology, York St John University, York, United Kingdom; Indian Institute of Technology Guwahati, INDIA

## Abstract

**Background:**

Individuals with Parkinson’s Disease (PD) often experience not only physical symptoms but also a higher prevalence of psychological issues, including depression, anxiety, schizophrenia, and psychotic symptoms, compared to the general population. The relationship between these physical and psychological symptoms remains poorly understood, particularly in relation to commonly used measures of physical function and a wider variety of psychological symptoms. Investigating this link is essential for optimising patient care. This cohort study is registered with OSF (https://osf.io/c7tvd).

**Objective:**

The primary aim of this study is to investigate the association between physical and psychological symptoms in PD. We will focus specifically on measures of physical function such as balance and mobility, assess the similarities between physiotherapist and participant-rated measures, and monitor changes over time.

**Methods:**

This cohort study plans to recruit 30 participants with PD, who will be monitored over a 12-month period. Data will be collected at baseline and completion, providing two data points per participant. Selected outcome measures will include physical function (objective measures), non-motor symptoms, participants perceived function, and engagement in physical activity. Correlations between outcomes will be analysed, and multivariate regression modelling may be employed for time-series analysis to evaluate how relationships change over time. Descriptive summaries of all outcomes will be presented in tables.

**Results:**

Ethical approval for this study was obtained on 15^th^ July 2024, with participant recruitment scheduled to begin in October 2024. Data collection is expected to conclude by August 2026, with final results anticipated within six months of study completion.

**Conclusions:**

This study will be the first to monitor this specific set of physical and psychological outcomes over time in individuals with PD. The findings are expected to provide valuable insights into the relationship between these symptoms, informing future research and potentially leading to larger-scale, multi-site studies conducted over extended assessment periods.

## 1.0 Introduction

Parkinson’s Disease (PD) is a multifaceted neurodegenerative condition impacting upon many aspects of an individual’s physical and psychological wellbeing. PD is the second most common neurodegenerative disease [1], and the most common neurodegenerative movement disorder worldwide [2], with prevalence tending to increase with age [3]. It is expected that by 2040, the global incidence of PD will exceed 12 million [4], significantly impacting healthcare systems [5]. It is therefore increasingly important to find innovative and forward-thinking approaches to improve treatments and to allow healthcare provision to keep pace with demand.

The three main physical symptoms of PD are tremor, muscle stiffness and slowness of movement [6]. These symptoms affect an individual’s ability to maintain their balance or ability to complete their usual activities of daily living (ADL’s), resulting in increased likelihood of falling and associated complications [7].

It is well documented that individuals with PD present with higher incidences of mental health problems such as depression, anxiety, schizophrenia and psychotic symptoms when compared to the general population [8, 9]. More specifically, up to 40% of people with PD (PwPD) will have depression [10] or anxiety [11], whereas this figure is only 17% in the general population [12]. Current NICE guidelines [13] do not address or provide specific recommendations for mental health problems in this population, instead referring to existing generic guidelines on depression in adults with chronic health problems and how to access allied health professionals (e.g., physiotherapists, PD nurse specialist). This is a striking contrast to other neurological conditions such as Multiple Sclerosis, where guidelines include specific recommendations for regular cognitive, emotional or mental health screening [14, 15].

Evidence in the elderly population suggests a relationship between physical and psychological presentations [16]. There is however a paucity of evidence to substantiate such a relationship in the PD population. It has been suggested that PwPD feel that anxiety may amplify their physical symptoms [17] and when they become more anxious the incidences of freezing of gait increase [18]. To add to this, a number of studies have suggested that as anxiety increases, so does the severity of motor symptoms as assessed by the Unified Parkinson’s Disease Rating Scale (UPDRS) [19–22]. Although these studies show that there might be an association between physical and psychological symptoms, this relationship is yet to be confirmed in more specific measures of physical function such as balance and mobility, or considering other psychological symptoms associated with PD [23, 24]. It is our belief that there is likely to be an intrinsic link between physical and psychological symptoms in PD which requires further investigation and monitoring over time.

Prior to proposing this study, a systematic review was completed as part of this ongoing PhD project [25]. The review found that despite a variety of physical and psychological outcomes commonly being collected together in PD research studies, only one study has explicitly examined the relationship between these outcomes [26]. More specifically, significant associations between physical functioning and psychological symptoms were found only in participant self-reported measures of function and not clinician-assessed measures. This suggests a potential mismatch between participant and clinician assessments which requires further investigation. Based on work by Essers et al. in individuals’ post-stroke [27], we hypothesise that symptoms of anxiety, depression, apathy and psychosocial functioning may be important in other neurological conditions, including PD, and will therefore be monitored within the planned study.

This review [25] highlighted the need for a more integrated mind-body approach to clinical and research practice, which can only benefit PwPD through more targeted assessment and treatment approaches considering physical and psychological symptoms simultaneously. We concluded that future research was required to uncover the true extent of any psycho-physical symptom interaction in PD, alongside considering the perceptions of PwPD regarding the impact of this on their daily lives.

Our meta-regression analysis using mean group-level data, completed as part of our previous study [25], showed a trend for the functional physical ability of PwPD to reduce as scores on depression outcomes increase, with a significant moderating effect of depression on gait, balance and transfer performance. This area of our research has been taken further through online surveys capturing the views of PwPD, carers, and physiotherapists, relating to potential psycho-physical interactions. Preliminary findings indicate that this area is recognised to be very important by all groups.

Given the high prevalence of anxiety and depression within PD, this study aims to further investigate the relationship between the physical and psychological components of participants clinical presentation. More specifically, this work aims to investigate whether there is any interaction between physical and psychological symptoms in PD, whether there are similarities between physiotherapist and participant-rated measures, and to monitor any changes in presentation over time. It is hoped that this study will act as a precursor to the completion of larger-scale multi-site research conducted over a longer assessment period.

The proposed study addresses a significant research gap regarding the interplay between physical and psychological symptoms in individuals with PD. While existing literature highlights the prevalence of mental health issues such as depression and anxiety among PD patients, there is a lack of comprehensive studies that explore how these psychological symptoms interact with specific physical manifestations over time. This study aims to fill this void by systematically monitoring both physical and psychological outcomes, thereby providing insights into their relationship and informing future clinical practices.

This study will be the first to evaluate the potential discrepancies between participant-reported and clinician-assessed measures of physical function and psychological health within this population over time. By exploring these relationships, the research seeks to uncover the intrinsic links between symptomatology that may inform future clinical practices and interventions. The findings are anticipated to not only enhance our understanding of psycho-physical interactions but also contribute to developing tailored treatment strategies that address both mental health and physical function in PD patients, ultimately filling a vital gap in current PD research and clinical guidelines.

The proposed research will collect data for selected physical and psychological outcome measures commonly used in clinical practice and supported by recommendations from the European Physiotherapy Guideline for Parkinson’s Disease [28]. Participants will be monitored for a period of 12-months, with data for each outcome collected at baseline and on completion, providing two data points per participant including baseline assessment.

Given the under-researched nature of this area it would seem that now is the ideal opportunity to investigate the potential interaction between physical and psychological symptoms in PD. This will be the first study monitoring this set of physical and psychological outcomes over time in PwPD. It is hoped that the outcomes of this research will provide an insight into the relationship between the physical and psychological symptoms of PD and help to inform future work aimed at developing our knowledge further. Should our findings align with the hypotheses below, we will take steps to confirm the findings across multiple sites and involving extended assessment periods.

It is hypothesised that:

1. Higher levels of anxiety, depression, apathy, and stress will be associated with reduced level of physical function.
2. Higher levels of physical function and lower levels of anxiety, depression, apathy and stress will be associated with higher quality of life.
3. Individuals with high levels of physical function will also report high levels of perceived function. Incidences of mismatch between patient-reported and therapist-assessed measures will be associated with participants reporting higher levels of anxiety, depression, and apathy.
4. Statements 1–3 will hold true throughout the assessment period.

Once understood in more detail, this work will provide a platform for future improvements in clinical provision for the benefit of patients and clinical services. The long-term impact may include recommendations for monitoring specific measures of physical function and psychological outcomes within research and clinical practice. Future work following completion of this study may also help to identify individuals who may be most ‘at risk’ of experiencing various symptoms so that a proactive approach can be taken to minimise the impact clinically.

## 2.0 Materials and methods

This cohort study is registered with OSF (https://osf.io/c7tvd). Following favourable opinion from the West of Scotland Research Ethics Service, Health Research Authority (HRA) approval to conduct this research was provided on 15/07/2024 (Reference: 24/WS/0078).

### Aim, design and setting

The primary aim of this study is to evaluate the potential interaction between physical and psychological symptoms in PD, assess whether similarities exist between physiotherapist and participant-rated measures, and to monitor any changes in presentation over time. Through data collection, an understanding of the clinical presentation of participants will be gained. Although limited by the amount of data we are able to collect at this stage, the question to be answered will be *‘what is the relationship between physical function and psychological symptoms in PD over time*?*’*. This will include evaluating whether similarities between physiotherapist and participant-rated measures exist, and the monitoring any changes in presentation over time.

This work will be completed as a cohort study, with physical assessments completed within the laboratory setting at York St John University, and questionnaire-based outcomes being completed by participants at home. As there is no intervention in this study, there is no need for participants to be divided into groups and will therefore be considered as one cohort.

Long-term, this project will help to increase the awareness of any psycho-physical interactions in PD. This may result in physical and psychological services working more closely together.

### Recruitment, selection criteria and sample size

Participants will be community-dwelling people living with Parkinson’s Disease. Potential participants will be identified from NHS services across the region, with primary recruitment via clinical caseloads within York and Scarborough Teaching Hospitals NHS Foundation Trust. Recruitment is scheduled to be completed between October 2024 and March 2025. Should recruitment not meet target, further recruitment is possible via local Parkinson’s UK networks if required.

**Fig 1:**
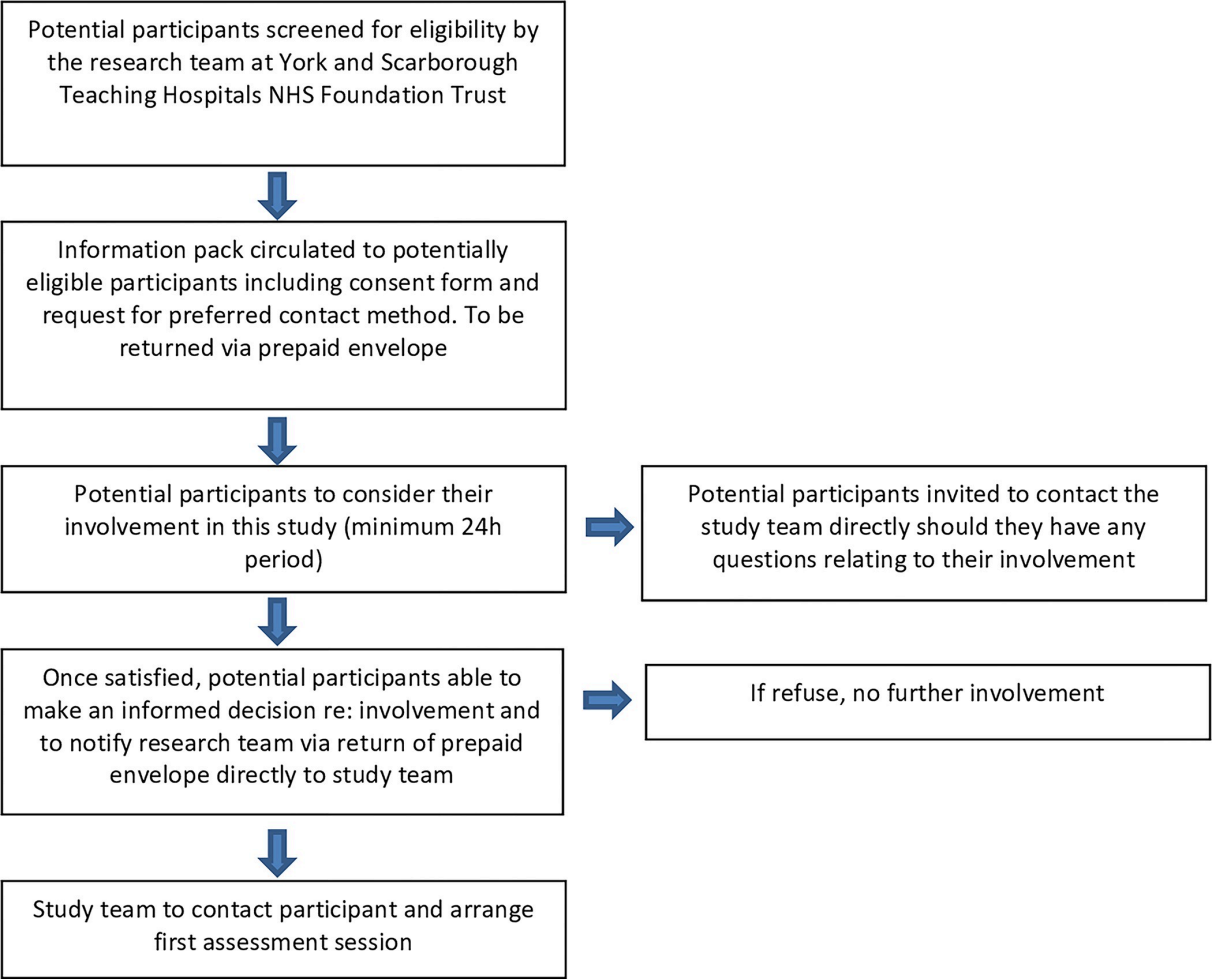
Flow chart detailing recruitment processes.

The research team at York and Scarborough Teaching Hospitals NHS Foundation Trust will identify potential participants via caseloads and clinical databases. They will contact identified individuals to discuss the study, and if accepted, provide the individual with a participant information pack. The information pack will contain an information sheet, consent form, and a form for participants to indicate their preferred method of contact which will be returned directly to the study team following full consideration. Once received the study team will contact participants to arrange the first assessment session. At this visit formal written consent will be gained via the consent form (S1 Appendix), and the first assessments will be completed. A flow chart detailing this process can be seen in Fig 1.

At all stages, potential participants will be considered for suitability against the following inclusion and exclusion criteria. Participants will be made aware throughout the recruitment process that as participation in the research is voluntary, they can withdraw consent at any time without giving any reason, and without their care or legal rights being affected.

Inclusion Criteria: i) Diagnosed with idiopathic PD as defined by the UK PDS Brain Bank Criteria [29]; ii) Aged 18 and over; iii) UK residents; iv) Capacity to give informed consent as stated in the 2019 HRA guidelines [30]; v) Sufficient understanding of English to be able to complete the required outcomes; vi) Able to attend the assessment sessions at YSJ University.

Exclusion Criteria: i) Non-idiopathic PD, due to potentially different mechanisms of action between types of PD [31]; ii) Any known premorbid neurological pathology (e.g. CVA) within their medical history, as this could introduce confounding variables [32]; iii) Unable to give informed consent; iv) Unable to attend the assessment sessions at YSJ University.

Our target sample size has been determined using sample size calculations based on the correlations between the Hospital Anxiety and Depression Scale (HADS) and MDS UPDRS Part II (motor experiences of daily living) [26]. Cross-sectional studies such as Still et al. (2021) can reveal the strength and direction of relationships between variables. By establishing these correlations, researchers can better estimate the effect size they expect to detect in a longitudinal study to observe significant changes over time. The study by Still et al., used different clinical outcomes to those proposed here, and was itself informed by recommendations from Bonett and Wright [33]. Within their research, a correlation (r = 0.575, p<0.05) was found between the HADS and the MDS UPDRS Part II (motor experiences of daily living), providing the best available estimate of the strength of any potential relationship between the proposed outcomes in our study.

G*Power [34, 35] is a statistical power analysis tool that helps researchers determine adequate sample sizes by specifying desired power levels, effect sizes, and significance levels for various statistical tests such as t-tests, F-tests, chi-square tests, and more. Using G*Power and the above correlation as an estimate, alongside an alpha level of 0.05 and power of 0.8, the required sample size was estimated at 21. We plan to recruit 30 participants to account for potential participant drop-out during the data collection phase.

Feedback on this proposed sample has been provided by a statistician working within York St John University. Should subsequent larger-scale research be undertaken, this study will inform revised sample size calculations based on standard deviation estimates for the outcomes included.

### Outcomes and materials

Information regarding the clinical presentation of participants will be gained, which will help to begin answering the question: *‘what is the relationship between physical function and psychological symptoms in PD over time*?*’*. This will include evaluating whether similarities between physiotherapist and participant-rated measures exist, and the monitoring any changes in presentation over time. Commonly used and clinically validated outcome measures will be used to assess this, and the planned outcomes can be viewed within Table 1. Where required, appropriate permissions to use each outcome measure have been obtained.

**Table 1:** Outcome measures included.

Physical	Non-Motor (Neuropsychiatric, Psychosocial, and Quality of Life)	Perception	Physical Activity
Modified Hoehn and Yahr (H&Y) Scale [36]	Scales for Outcomes in Parkinson’s Disease–Psychosocial Functioning (SCOPA-PS) [37]	Falls Efficacy Scale–International [38]	International Physical Activity Questionnaire–Elderly (IPAQ-E) [39]
Timed-up-and-go (TUG) [40]*	Hospital Anxiety and Depression Scale (HADS) [41]	Walk-12G Questionnaire [42]	
6-minute walk (6MWT) [43]*	Apathy Evaluation Scale–Self Report [44]		
Five times Sit to Stand [45]*	EQ-5D-5L [46]		
Berg Balance Scale [47]			

*Selected physical outcome measures will also include additional data collection as below

1. 6-minute Walk Test (6MWT): In addition to standard completion, change in walking speed monitored over time via time taken per 60m lap. Time per lap during the 6MWT will be recorded for each participant. This will be done using a stopwatch and recording the time taken per lap. Time per lap will be used to estimate participants walking speed for each lap, which will in turn be used to assess any changes over the course of the 6-minute test. This will provide increased insight into participants functional ability and endurance over and above the total distance walked.

2. Timed-up-and-go (TUG) Test: Alongside standard completion, a Qualisys motion capture system consisting of up to 10 cameras and markers placed on participants feet will be used to record participants step length and width, cadence, velocity, and circumference of turning arc during completion of the TUG. This will give more information regarding some of the factors that may contribute towards TUG performance.

3. Five times Sit-to-Stand Test: Further to standard completion, a Qualisys motion capture system (as above) and markers placed on participants hips will be used to record participants velocity and power during the sit-to-stand task. Forces in effect during the task will be recorded via a force plate which the participant will be stood on for the duration of the task. As pervious, this will provide a higher level of insight into task completion than given by the time taken to complete the task in isolation.

Where applicable, reflective markers will be placed over the top of clothing/footwear, however prior to marker placement, participants will be asked about any allergies.

Physical outcomes will be completed in the following sequence in order to minimise any impact of fatigue: Modified Hoehn and Yahr Scale; Timed-up-and-go; Five times Sit to Stand; Berg Balance Scale; and 6-minute walk test. Participants are able to complete questionnaires in any sequence they wish. Participants will be requested to provide updates on any changes in the medical management of their condition.

### Study processes and study timeline

As there is no intervention in this study, there is no need for participants to be divided into groups and will therefore be considered as one cohort. Once recruited and screened for eligibility, each participant will visit the laboratory on two occasions.

Visit 1 = Baseline Assessment of clinical physical outcomes (Approx 90 mins); Visit 2 = Follow up assessment of clinical physical outcomes (Approx 90 minutes). All questionnaire-based outcomes will be completed by the participant at baseline and on completion. This is expected to take approximately 60 minutes to complete on each occasion. Physical outcomes will be completed by trained clinical personnel. Questionnaires will be provided by trained clinical personnel, with participants completing the self-report measures allowing us to capture comprehensive psychological profiles. Signposting to appropriate services will be provided in case of any distress experienced, as per our distress protocol.

Psychological symptoms such as depression, anxiety, stress, and apathy will be assessed by asking participants to complete questionnaires at home and will take approximately 60 minutes to complete. Participants will be asked to return questionnaires via pre-stamped and addressed envelopes. Physical assessments of balance, mobility and physical endurance will be assessed at York St John University and will take about 90 minutes to complete including periods of rest. All physical and psychological assessments will be repeated at 12-months, and physical assessments completed by the same individual at both timepoints for all participants.

In total each participant would be asked to commit approximately five hours of their time to assessments over a 12-month period. Visiting the laboratory on two occasions is a potentially significant burden for both participants and carers who may be required to assist with travel. Based on PPI completed it is felt that this would be acceptable to potential participants. Participants will be made aware that they are free to withdraw without consequence at any stage during data collection, however data collected prior to that point will be retained for analysis.

**Fig 2:**
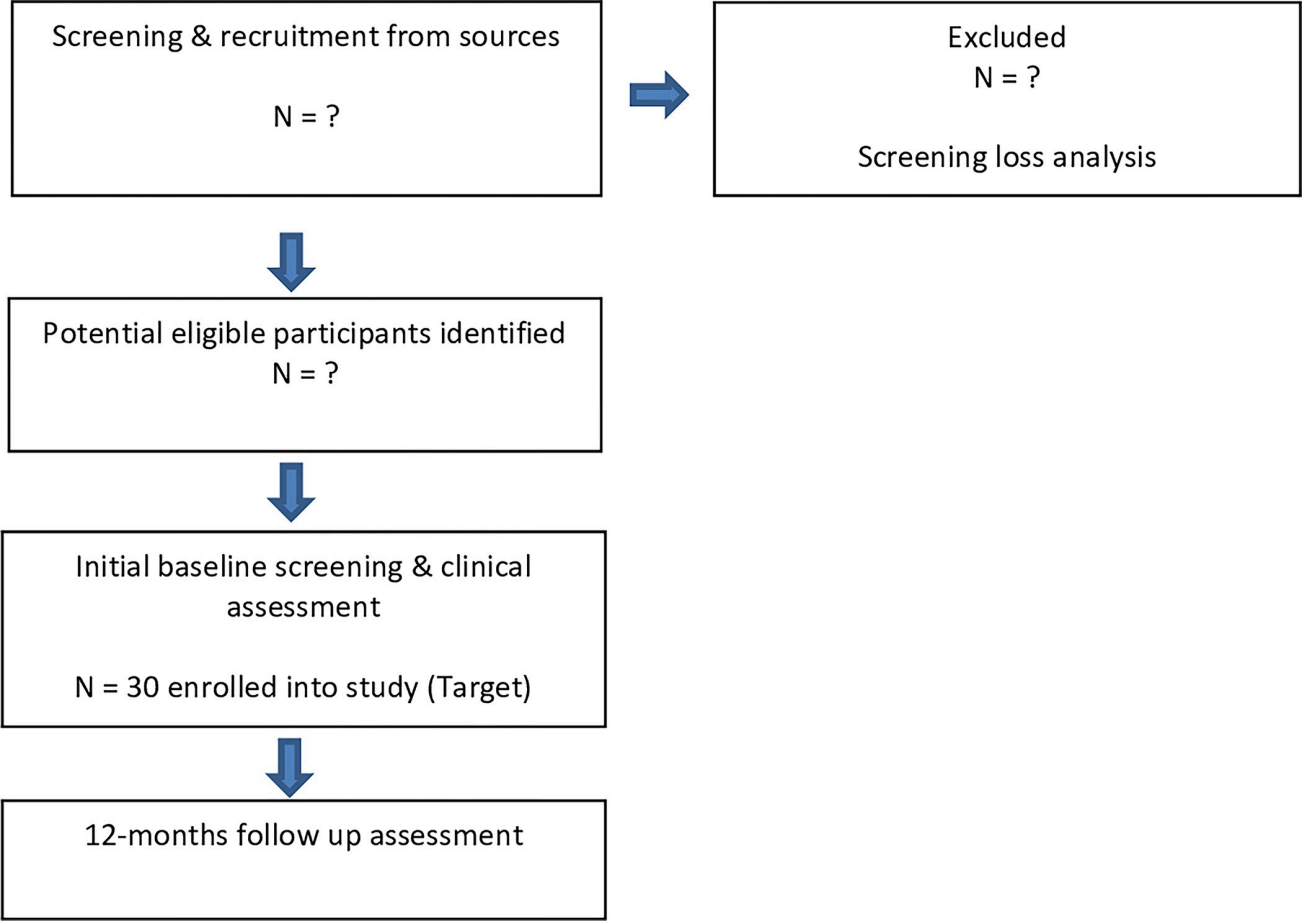
Flow chart detailing overall study processes.

**Fig 3:**
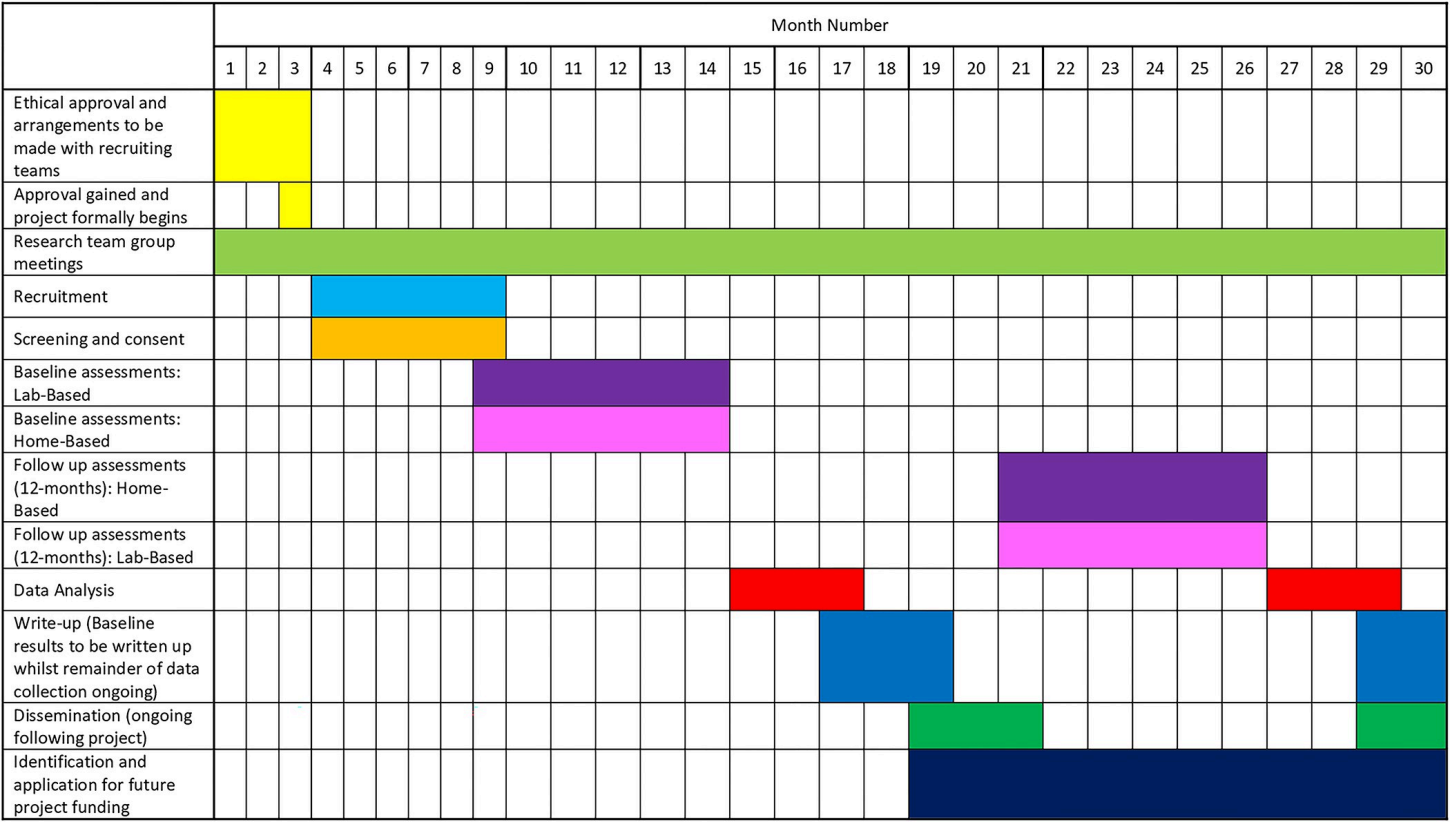
Overall estimates of project timescales.

Participants will be requested to provide updates on any changes in the medical management of their condition, however this will not automatically exclude them from participating, and will be acknowledged at follow-up. Please see flow chart in Fig 2 for details of the overall study processes. Overall estimates of project timescales can be seen in Fig 3.

### Data management and availability

All members of the research team will have completed and be up to date with GCP training. All personal data will be handled with extreme care and will adhere to the Data Protection Act and in accordance with MRC guidelines for the handling of personal data. Data collection and storage will be undertaken in accordance with; The Caldicott Principles, Data Protection Act 2018 and Confidentiality: NHS Code of Practice 2003.

Participants will be allocated a number following recruitment, and data anonymised following collection. Only the project team aware of which number relates to each participant for the purpose of ensuring consistency during follow-up assessments. Data will be stored in an excel spreadsheet and thereafter will be inputted on SPSS and analysed accordingly. All data files will be password protected files stored in the YSJU password protected One: drive account owned by PH.

All data will be handled securely and in accordance with the data management plan submitted as part of the application for ethical approval. RaY and RaYDaR will be used as data repositories to publish data linked to this project and will be made available as soon as practical following completion.

### Data analysis

At each time point and for each variable combination the following analysis will be completed. If the data is found to be normally distributed then parametric tests such as Pearson’s r will be used, however if this is not the case then non-parametric equivalents such as Spearman’s Rank Correlation will be used. If appropriate, multivariate regression modelling will be used to complete a time-series analysis to evaluate how any relationship between outcomes and covariates (such as age, disease duration, comorbidities, and medications) changes over time. Descriptive summaries will be presented in tables for all outcomes measures.

We plan to analyse variations in symptom presentation through participant-reported outcomes and clinical assessments, allowing us to explore how these differences may influence psycho-physical interactions over time. Missing data will be handled using multiple imputation techniques, which will allow us to estimate missing values based on available data while preserving the integrity of our analyses. Additionally, we will conduct sensitivity analyses to assess the impact of missing data on our findings.

Data collected as part of this study may be used to assist in calculating the sample size for a future larger scale multi-site study. This will include establishing which of the outcomes assessed are most appropriate to be the primary outcome of any larger-scale study and whether any other factors should be considered.

### Safety and ethical considerations

The study will be conducted in full conformance with principles of the “Declaration of Helsinki”, Good Clinical Practice (GCP) and within the laws and regulations of the country in which the research is conducted.

All physical assessments will be conducted in a safe environment and under the supervision of a qualified physiotherapist. Each measure is low cost in comparison to other methodologies, with a low participant burden given the short time taken to complete. Adequate rest periods will be provided between each physical assessment with participants made aware they are in control of the level of physical exertion reached.

Within the proposed study there is a potential participant burden associated with of monitoring symptoms without direct treatment aimed at specific areas of difficulties. It is hoped that participants will be motivated to engage with the project with a view to understanding PD and potential symptom interactions, however it is understood that a large drop-out rate or difficulty recruiting is likely to indicate a significant burden.

Consideration has been given to each of the following ethical issues: Confidentiality, Informed consent, Anonymity, Persuasion and pressure, and Failure to disclose interest.

Confidentiality: In this project, confidentiality will be maintained throughout. All information provided by participants including any personal data will be stored safely and only accessible by the study team. Potential participants will be made aware of confidentiality arrangements within the Participant Information Sheet (S1 Appendix).

Informed Consent: Prior to completing the study, participants will be directed towards and encouraged to read the Participant Information Sheet. The PIS will include a full description of the study aims, objectives and methodology, with individuals encouraged to contact the study team should they have any questions prior to beginning their participation.

Anonymity: No personally identifiable information will be stored following completion. Should participants wish to be updated on future work they will be encouraged to provide an email address however this will not be used alongside data recorded or for other purposes aide from updating participants as described.

Persuasion and pressure: It will be made clear on the Participant Information Sheet that individuals are under no obligation to complete the study and are free to discontinue their participation at any stage. Participants will have the opportunity to decline any assessment they do not wish to complete.

Failure to disclose interest: The Participant Information Sheet will disclose any competing interests for all members of the research team.

It is felt that the present study does not pose any significant ethical concerns, with any potential areas of concern addressed as above.

## 3.0 Discussion

The above sections have addressed the need to complete this research and the proposed methodology. This section will address identified study limitations, dissemination plans, study monitoring and termination, and PPIE completed during development, before providing a short summary of the work from an overarching perspective.

### Study limitations

Despite our attempts to cover a range of motor and non-motor assessments within this study, any relationship between physical function and psychological symptoms in PD is likely to be highly complex and include factors that we are unable to control for or monitor within this work. This also limits any attempts to monitor how any relationship between physical function and psychological symptoms develops as the condition progresses. We acknowledge this limitation and will report this alongside the outputs of any modelling we are able to produce based on the data collected.

A further limitation includes our decision to exclude participants with non-idiopathic PD, potentially limiting our understanding of the wider topic. We made this decision to focus specifically on idiopathic PD to minimise variability in disease mechanisms and symptomatology, which could complicate our analysis. However, we acknowledge that including other PD variants could enrich our understanding of psycho-physical interactions and will consider this for future research which will include a larger sample size.

### Dissemination plans

All participants will be offered the opportunity to receive a copy of the findings and a personalised report detailing their results throughout the study period. We will also offer a separate online forum to disseminate and discuss the findings with PwPD, carers, and clinicians involved in the project. Further sessions with interested individuals/groups will be organised via Parkinson’s UK.

Philip Hodgson (Lead Researcher) is treasurer for the Chartered Physiotherapists in Mental Health network. The findings of this study will be presented within their forums, conferences/events, and website. Dr. Charikleia Sinani leads the Consortium Yorkshire and the Humber Hub of the Council for Allied Health Professions Research (CAHPR). The findings will be included on its website and presented at the appropriate events organised by the network (e.g. workshops, seminars, conferences). Findings will be disseminated more widely through the CAPHR and CSP appropriately. A final report will appear on the CSP website and be submitted for presentation at relevant conferences such as the CSP annual conference and World Parkinson’s Congress.

We are planning to produce a leaflet for clinicians, PwPD and carers aimed at raising awareness of the psychological symptoms of PD and publicising the research we are conducting in this area. Our hope is that this will encourage clinicians to assess and act upon any psychological symptoms identified, including making onward referrals to appropriate services. We will distribute copies of this leaflet to the CSP, all above groups and NHS partners. We intend to submit findings from this study to a peer reviewed journal such as ‘Physical Therapy’ which engages and inspires an international readership and has published studies with similar designs. Lastly, all findings from this research will be used to support the submission of a larger scale study to the NIHR Research for Patient Benefit Scheme (RfPB).

### Study monitoring and termination

Professor Divine Charura (Primary PhD Supervisor) School of Education, Language and Psychology, York St John University, Lord Mayor’s Walk, York, YO31 7EX. Email: d.charura@yorksj.ac.uk), will monitor and audit the conduct of the research, and will review interim safety and efficacy data from the trial.

The trial will be suspended and/or stopped if there is clear evidence of harm. This will be decided by the PI in conjunction with academic supervisors, sponsor and REC. Should the study be terminated early, the investigative team will decide which study objectives can be addressed in an unbiased manner with the data available.

### PPIE completed

Plans for the proposed research were taken to Parkinson’s UK volunteers including PwPD and carers for PwPD. Seven volunteers; including some who had been involved in guiding previous project phases such as the design of surveys discussed previously; provided comments and suggestions about aspects including the perceived acceptability of the research design, methodology, clarity of research idea, purpose, and potential barriers to completion.

Volunteers reported that they perceived this area as important in improving our understanding of PD and foresee a significant impact on care/treatment if this understanding is developed. Volunteers reported that the cohort study design, including the planned outcomes and number of visits were feasible and realistic if adequate rest periods were provided. Feedback provided has directly informed the design of this proposed study, such as the inclusion of rest periods and the option to complete paper-based questionnaires from home.

### Summary

We currently know very little about the relationship between physical functioning and psychological symptoms in PD. This study aims to improve our understanding of the relationship between physical functioning and some of the most common psychological symptoms of PD, and how this may change over time.

Ultimately this research aims to inform changes in research and clinical practices and will encourage the monitoring of specific physical and psychological outcomes within interventional studies. Larger-scale work also has the potential to provide an insight into what stage(s) of Parkinson’s individuals may be most ‘at risk’ of experiencing various symptoms, and any changes in physical and/or psychological symptoms that may help to identify future issues before these arise. This will help to build upon current subtyping practices based on clinical symptoms and data-driven neuroimaging [48–51].

Depending upon the strength and direction of any relationship, this is likely to provide a rationale for increased focused on improving symptom recognition, management, and interventions provided by physiotherapists. This increased focus will include a proactive approach to identifying which patients are most likely to benefit from a targeted physiotherapy approach alongside onward referrals to other services. We believe that integrating our results into clinical practice could lead to the development and wider use of tailored interventions that address both mental health and physical function in PD patients.

The team involved in this project are well-placed to complete this work, develop subsequent future research, and to work with policy makers to ensure that findings from a larger study have the potential to influence practice.

## Supporting information

S1 Appendix Participant information sheet and consent form (see supplementary file S1 Appendix). (PDF)
