# Supplementary material for: Psycho-physical interactions in Parkinson’s Disease: Protocol for a prospective observational cohort study
Source: PLoS One. 2024 Dec 16;19(12):e0315345. doi: 10.1371/journal.pone.0315345 (PMC11649133; doi:10.1371/journal.pone.0315345)
Supplement: S1 Appendix — (PDF) [file pone.0315345.s001.pdf]

Participant Information Sheet

Version 1.2
Updated 15/07/2024

Title of Project	**Psycho-Physical Interactions in Parkinson's Disease: 12-month Cohort Study**
**Researcher name and contact details**	Philip Hodgson<br>*PhD Student*<br>*School of Science, Technology and Health*<br>*philip.hodgson@yorksj.ac.uk*
**University contact details**	York St John University, Lord Mayors Walk, York YO31 7EX. Tel: 01904 624624

Invitation and Brief Summary:
You are being invited to take part in a research study. Before you decide whether or not to participate it is important that you understand why the research is being done and what it will involve. Please take the time to read the following information carefully, and feel free to ask if anything is unclear.

Part 1: Project Details

**Introduction**	Individuals with Parkinson's commonly experience psychological symptoms alongside the physical symptoms of their condition, with rates of various psychological diagnoses greater than in the general population.<br><br>At the moment we don't know much about the potential interaction between these physical and psychological symptoms in Parkinson's, or how any relationship between symptoms may change over time.<br><br>This project is being completed as part of a PhD research project undertaken by Philip Hodgson. Philip is an NHS Physiotherapist completing this research part-time alongside his clinical work. The project is supervised by Professor Divine Charura, Dr Alastair Jordan, and Dr Charikleia Sinani at York St John University. Please contact Philip via email should you have any questions.
**What is the purpose of the project?**	This study aims to explore how various measures of physical function relate to psychological presentation in people living with Parkinson's.<br><br>We are hoping to recruit a total of 30 individuals with Parkinson's who are willing to complete a series of motor and non-motor assessments on two occasions 12-months apart.
**What can I expect?**	All participants will be asked to complete the same series of assessments at two timepoints:<br>• Baseline (first assessment)<br>• 12-months<br><br>At each timepoint you will be asked to complete some physical and questionnaire-based outcome measures. Physical assessments will be completed at York St John University and take approximately 90-minutes (inclusive of rest periods), with questionnaires taking approximately 60-minutes and being completed at home.<br><br>Physical outcomes will involve things such as assessments of your balance and mobility, through tasks such as standing up, turning, and walking. Some of these assessments will require a small number of markers to be placed on top of your clothing to record information

	about how tasks are completed. These markers are non-invasive and are easily removable without damaging clothing. If you are unhappy to complete any task, please let the investigator know and you will not be asked to complete it.<br><br>Questionnaires will be used to assess how confident you feel during certain tasks, assess symptoms such as anxiety and depression, and record information about your level of physical activity.<br><br>In total you will be asked to commit approximately 5 hours of your time to assessments over a 12-month period.
**Where will assessments take place?**	Physical assessments will be completed at our laboratory at York St John University, Haxby Road campus (YO31 8TA). You will be entitled to 4 hours free parking on site and disabled parking spaces are available if required. The laboratory is on the ground floor and there is lift access to a café area. Toilets, including disabled facilities are available.<br><br>Questionnaires are intended to be completed at home and returned to the research team via post using a pre-paid envelope which will be provided. Should you choose to, you are welcome to complete these in the café area on site.
**Why have I been asked to take part?**	You have been asked to participate in this study to help improve our understanding of the relationship between physical and psychological symptoms in Parkinson's.<br><br>A member of staff at York and Scarborough Teaching Hospitals NHS Foundation Trust has identified you as someone who potentially meets the criteria to participate and has sent this information sheet to you following an initial conversation with you.<br><br>You must meet the following criteria to participate in this study:<br>i. Diagnosed with idiopathic Parkinson's Disease<br>ii. Aged 18 and over<br>iii. UK resident<br>iv. Have capacity to give informed consent to participate<br>v. Able to attend the assessment sessions at YSJ University.
**Do I have to take part?**	Participation in this study is voluntary and will not affect any other aspects of your clinical treatment.<br><br>Even if you initially agree to participate in the study, you are free to withdraw at any stage during data collection by informing one of the researchers involved in the study. Any data collected prior to that point will be retained for analysis.<br><br>All data collected will be pseudonymised by the use of a participant number, ensuring that your personal identity is protected. This means that while your data will be linked to a unique participant number, it will not be directly identifiable without access to the key that links participant numbers to personal information. The results of the study may be published for scientific purposes; however, your identity will not be revealed.<br><br>You may wish to discuss your involvement in this study with your GP, hospital consultant, or nurse specialist prior to enrolling.
**What are the possible benefits of taking part?**	Since there is no treatment offered as part of this study, we do not expect there to be any direct medical benefits of taking part in this study. Participants often find value in contributing to scientific research and we hope that this will be an enjoyable experience for you. By participating, you are contributing to important research that may help improve understanding and treatment of Parkinson's.
**Are there any risks?**	This study monitors your symptoms over time and does not provide any treatment. Participating in this study does not impact on any other treatment you may receive.

	Physical assessments involve a small level of risk however you will be provided with adequate rest periods and be able to decline any assessments that you do not feel able/comfortable to complete. Although there are no major risks, we acknowledge that participating in this study requires a significant time commitment to complete the various assessments over a 12-month period.<br><br>**Due to the nature of assessing psychological symptoms, this may include topics that you find sensitive. Should you require any physical or psychological support during or following this research please contact your local GP, NHS 111 service, or relevant charity organisations such as Parkinson's UK via email at hello@parkinsons.org.uk or telephone 0808 800 0303 (helpline) and MIND via email at info@mind.org.uk or telephone 0300 123 3393 (helpline).We have a specific distress protocol in place for this project therefore please inform the research team if you require any additional support.**
**How will we use information about you?**	We will need to use information from you for this research project. This information will include your name and contact details. People will use this information to do the research or to check your records to make sure that the research is being done properly. People who do not need to know who you are will not be able to see your name or contact details. Your data will have a code number instead.<br><br>We will keep all information about you safe and secure. Once we have finished the study, we will keep some of the data so we can check the results. We will write our reports in a way that no-one can work out that you took part in the study.
**What are your choices about how your information is used?**	You can stop being part of the study at any time, without giving a reason, but we will keep information about you that we already have. We need to manage your records in specific ways for the research to be reliable. This means that we won't be able to let you see or change the data we hold about you.
**Where can you find out more about how your information is used?**	You can find out more about how we use your information<br>• at www.hra.nhs.uk/information-about-patients/<br>• our leaflet available from www.hra.nhs.uk/patientdataandresearch<br>• by asking one of the research team<br>• by sending an email to us@yorksj.ac.uk<br>• by ringing us on 01904 624 624
**What information will be collected?**	In addition to recording scores from the various assessments completed, we will also record your name, telephone number, email and postal addresses. This personal information is purely for the purpose of arranging assessment sessions and will be stored separately to your scores.
**Who will have access to the information?**	All information that we collect about you during the study will be kept strictly confidential and stored securely. Results from this study will be published in academic journal(s) and may be used when presenting the work at conferences. You will not be identifiable in any publications of the results or reports from this study.<br><br>If you decide to take part, we will collect some personal information about you including your name, address and contact details. Authorised members of the research team will hold this data and store it securely so they can contact you for the purpose of this study only. All information processing will be completed in the UK and will comply with General Data Protection Regulations (GDPR).<br><br>Anonymised information from this study may be shared with other researchers doing similar research in the future. None of your personal identifiable information will be shared, and you will not be identifiable from this data.<br><br>If you decide to take part, your GP will be informed of your participation via letter however will not have access to any data collected.

**Where will the information be stored?**	All data will be stored in the York St John One Drive secure system. People will use this information to do the research or to make sure that the research is being done properly. We will keep all information about you safe and secure. Data will be disposed according to the UK Data Archive 'Managing and Sharing Data - best practice for researchers' guide (3rd edition). This document can be found here: https://ukdataservice.ac.uk/media/622417/managingsharing.pdf
**How long will the information be retained?**	Personal information will only be retained for as long as it is necessary up to a maximum of 3 months following the end of the study. In the interest of transparency in research, the pseudonymised data will be deposited in York St. John University's data repository (RaYDaR) and made publicly available.
**Ethical information**	Ethical approval to conduct this research has been provided by West of Scotland Research Ethics Service (Reference: 24/WS/0078), in accordance with its ethics review and approval procedures.
**Insurance and indemnity**	This research study is sponsored by York St John University. The sponsor holds appropriate insurance policies that apply to this study. These policies cover any potential harm that may come to you as a result of your participation in this research.
**What happens next?**	**Thank you for your interest and for reading this information.**<br><br>Please consider all information provided within this information sheet and contact a member of the research team using the contact details above if you have any further questions.<br><br>If you are happy to participate in this study, please complete the attached contact form and a member of the study team will be in touch to discuss the next stages of your participation. This contact will take place via your stated preferred method.<br><br>Any person considering participation in this research project, or agreeing to participate, may raise any questions or issues with the researchers at any time. In addition, any person not satisfied with the response of researchers may raise ethics issues or concerns and may make any complaints about this research project by contacting Dr Charlotte Haines-Lyon, Chair of the Ethics Committee for the School of Education, Language and Psychology' (Email: c.haineslyon@yorksj.ac.uk). All research participants are entitled to retain a copy of any Participant Information Form and/or Participant Consent Form relating to this research project.

Part 2: Privacy Notice

All personal information gathered and held by York St John University (detailed in Part 1 of this Participant Information Sheet) is treated with the care and confidentiality required by the UK General Data Protection Regulation (UK GDPR) and the Data Protection Act 2018. For the purposes of processing your personal information, the data controller is York St John University. The University's Data Protection Officer is the PVC Governance and Student Life.

**Legal basis for processing your information and additional condition for processing special category data**	**Task in the public interest.**<br><br>Universities and the NHS are funded from taxes and they are expected to do research as part of their job. They still need to be able to prove that they need to use patient data for the research. In legal terms this means that they use patient data as part of 'a task in the public interest'.<br><br>If they could do the research without using patient data they would not be allowed to get your data.<br><br>Researchers must show that their research takes account of the views of patients and ordinary members of the public. They must also show how they protect the privacy of the people who take part. An NHS research ethics committee checks this before the research starts.
**Your rights in relation to personal data**	Under the GDPR, you have a right to:<br>• be kept informed as to how we use your data;<br>• request a copy of the data we hold about you via a Subject Access Request;<br>• update, amend or rectify the data we hold about you;<br>• change your communication preferences;<br>• ask us to remove your data from our records;<br>• object to or restrict the processing of your information<br>• raise a concern or complaint about the way in which your information is being used.
**Any questions of concerns?**	If you have any questions or concerns about the way we are collecting and using your personal data we request that you contact the University by emailing: gov.compliance@yorksj.ac.uk. You also have the right to complain to the Information Commissioner's Office (ICO) about the way in which we process your personal data. Details can be found at: https://ico.org.uk.

**Title of Project**	**Psycho-Physical Interactions in Parkinson's Disease: 12-month Cohort Study**
**Researcher name and contact details**	Philip Hodgson<br>*PhD Student*<br>*School of Science, Technology and Health*<br>*philip.hodgson@yorksj.ac.uk*
**University contact details**	York St John University, Lord Mayors Walk, York YO31 7EX. Tel: 01904 624624

Part 3: Participant Consent	Please initial box	
	Yes	No
1. I confirm that I have read the information sheet dated .... (version............) for the above study. I have had the opportunity to consider the information, ask questions and have had these answered satisfactorily.		
2. I understand that my participation is voluntary and that I am free to withdraw at any time during data collection without giving any reason, without my medical care or legal rights being affected. I understand that data collected prior to withdrawal will be retained for analysis.		
3. I understand that relevant sections of my medical notes and data collected during the study, may be looked at by individuals from the study team and York St John University, where it is relevant to my taking part in this research. I give permission for these individuals to access to my records.		
4. I am happy for the results obtained from my participation to be used within future journal publications and conference presentations.		
5. I agree to my General Practitioner being informed of my participation in the study.		
6. I understand what the information I provide will be used for, how it will be stored and how long it will be retained.		
7. I agree to take part in the above study.		

**Name of Participant**	
**Signature**	
**Date**	

**Name of Researcher**	
**Signature**	
**Date**	
